# The integration and associated challenges of Mental Health Competencies in Undergraduate Nursing Education: a scoping review

**DOI:** 10.1186/s12912-025-02942-z

**Published:** 2025-03-28

**Authors:** Precious Chibuike Chukwuere, Nombulelo Esme Zenani, Katlego Mthimunye, Rosemary Godbold, Ghada Shahrour

**Affiliations:** 1 NuMIQ Research Focus Area, Faculty of Health Sciences, North-West University, Potchefstroom, South Africa; 2 NuMIQ Research Focus Area, Faculty of Health Sciences, North-West University, Mahikeng, South Africa; 3 Department of Nursing, Midwifery and Health, Faculty of Health and Life Sciences, Northumbria University, Newcastle upon Tyne, UK; 4 School of Health and Social Work, University of Hertfordshire, Hatfield, Hertfordshire UK; 5 Department of Community and Mental Health Nursing, Faculty of Nursing, Jordan University of Science and Technology, Irbid, Jordan

**Keywords:** Competencies, Nursing education, Integration, Mental health, Scoping review, Undergraduate

## Abstract

**Background:**

Educational institutions play a pivotal role in meeting healthcare needs by educating future professional nurses and other healthcare professionals. However, nursing education encounters challenges such as insufficient competencies among undergraduates and a gap in theory-practice integration in the curriculum. This scoping review aimed to map out the existing literature on integrating Mental Health Competencies in undergraduate Nursing Education and associated challenges.

**Methods:**

The electronic databases of CINAHL, MedLine “PubMed”, Scopus, Science Direct, and Emerald Insight were searched for peer-reviewed articles on the subject, utilizing English search terms. Two authors independently reviewed the identified articles that met the inclusion criteria. The screening and selection process was conducted in the following phases: Firstly, the search results were imported into EPPI reviewer software, and duplicates were removed using the software’s built-in function. Secondly, careful screening of the titles and abstracts of all imported studies was followed based on the inclusion criteria. Thirdly, the reference list of the identified articles was screened to foster a comprehensive coverage of evidence. Full-text screening was conducted for all the identified articles, and the research team carefully scrutinized this process.

**Results:**

The initial literature search generated 717 articles. Upon identification and careful screening, 17 eligible articles met our inclusion criteria. Two key themes were reported: Integrating Mental Health Competencies in Undergraduate Nursing Education and Challenges to integrating Mental Health Competencies in undergraduate Nursing Education.

**Conclusion:**

The findings of this scoping review indicated that various efforts are being made toward integrating mental health nursing into undergraduate nursing education. However, these efforts are constantly confronted by different challenges, such as societal stigma, patient behaviours, unequal student contributions in group work activities, and difficulties in understanding patients’ symptoms. Meaningful efforts should be made towards addressing these challenges to prepare future nurses with the necessary mental health competence.

## Introduction

Educational institutions play a pivotal role in meeting healthcare needs by educating future professional nurses and other healthcare professionals [1]. However, nursing education encounters challenges such as insufficient competencies among undergraduates [2] and a gap in theory-practice integration in the curriculum [3, 4]. Mental health education is one of the critical aspects of nursing education that requires attention. It is integrated into nursing education at various levels across different countries to equip students with the necessary knowledge and skills for future practice [5–7]. Globally, different countries prepare nurses with mental health knowledge at various levels. For instance, in the United States, mental health knowledge is taught at the basic level for nurses preparing for registration as registered nurses. The nursing students, after graduation, are expected to possess foundational knowledge and skills to care for patients with mental illnesses [8]. In contrast, the United Kingdom (UK) adopts a distinct approach where nursing students are expected to choose from four distinct fields; adult, child, learning disability, and mental health during pre-registration [9, 10]. However, it has been proposed that a general approach to nurse education, where specialization occurs only after registration, should be adopted to align with international standards [10]. In countries such as Pakistan, Brazil, and South Africa, mental health pedagogy is integrated at the undergraduate level [5, 11, 12].

Notwithstanding these variations in nursing education, a common challenge of adequate preparation of students with mental health competence in generalist nursing programs remains persistent. It has been reported that undergraduate nursing students in such general training programs often receive limited preparation in mental health education, and insufficient depth in the curriculum in the aspect of mental health content [13]. For example, Olmos et al. [11] reported on the historical evolution of nursing education in Brazil, noting advancements in psychiatric nursing teaching over time, including the inclusion of psychoanalysis and the rise of psychiatric nursing theorists. However, teaching methodologies remained traditional and inflexible, suggesting a gap in adapting teaching methods to evolving understandings of mental health care. In the UK, concerns have been raised regarding the increasingly generic nature of nursing curricula and the extent to which mental health competencies are emphasized [9]. In Saudi Arabia, most mental health nursing courses, including theoretical and practical components, are taught in the final year of undergraduate nursing education. However, among the students, mental health clinical placements are not well accepted, and the anxiety and stress they experience could influence the quality of their clinical learning experience [14]. Atashzadeh-Shoorideh et al. [15] and Lockertsen et al. [16] established that the lack of adequate content in mental health curricula is a challenge that underscores the speculation that undergraduate nurses are not well prepared to manage mental health care among patients. Similarly, Bennett [17] asserts that the World Health Organization and other health agencies have reported that many nurses are not adequately prepared at the undergraduate level to provide mental health care to patients. Undergraduate nursing students sometimes struggle with translating theoretical knowledge into practice [18]. This is attributed to inadequate quality of theoretical and experiential learning opportunities, issues of variation, and inconsistencies in representing mental health in the undergraduate nursing curriculum [13]. Also, a theory-practice gap exists [19, 20], whereby undergraduate nursing students struggle with grasping theoretical concepts and their application in practice [18]. Furthermore, negative attitudes of nurses in clinical practice make it difficult for nursing students to develop mental health competence and, subsequently, provide mental health care [18, 21]. This scoping review explores how various general nursing programs integrate mental health competence and the associated challenges in integrating it.

Mental health competence encompasses the knowledge, skills, and attitudes necessary to effectively understand, assess, and address mental health illnesses [22]. It includes basic knowledge of mental health disorders, and causes, including their impacts on an individual, family, and the community. Furthermore, these competencies include the ability to conduct mental health assessments and apply diagnostic criteria, including implementing evidence-based intervention and establishing therapeutic relationships while delivering person-centered and compassionate care [22–25]. Drawing from Watson’s Caring Science theory which emphasizes holistic and compassionate care, these competencies integrate the mind, spirit, and body into the caring process, advocating for an empathetic, person-centered approach [26]. Through the application of Watson’s Caring theory, in this scoping review, mental health competence highlights the creation of a healing environment crucial to mental health. Given the increasing demand for mental health services, integrating mental health competence into undergraduate nursing education is crucial for the adequate preparation of future nurses with the necessary knowledge and skills to care for individuals with mental illnesses [5]. This requires a comprehensive approach that addresses various aspects of nursing, such as curriculum and integration, identification of core competence, didactic instruction, clinical experience, simulation training, interprofessional education, cultural competence, Mental Health First Aid (MHFA), and evidence-based practice [27–29]. Nurses play a frontline role in mental healthcare, including mental health promotion, prevention of mental illnesses, and providing care for individuals with mental illnesses [17, 30]. Therefore, it is essential for undergraduate nursing education programs to integrate mental health competencies into their curricula and teaching approaches to meet the demands of mental healthcare [31, 32]. Mental health competence requires a systematic approach that incorporates theoretical knowledge and an understanding of critical components of care [33]. Research has shown that nurses with higher competence can better apply their skills in mental health nursing and other areas of clinical practice [34, 35]. The World Health Organization has emphasized the need for mental health education in nursing curricula and recommends incorporating mental health components in undergraduate and post-graduate programs [36]. By integrating mental health competencies, we can address the issue of low competence among mental health nurses [37], and ensure adequate preparation of nurses to effectively address the rising mental health needs of individuals and communities [38, 39].

Developing mental health competence among student nurses requires interprofessional education and collaborative practice [40] and integrating integrated behavioural health and primary care skills and knowledge into undergraduate and graduate curricula [41]. It also requires teaching undergraduate nursing students about self-care and incorporating technology into nursing education [42]. Additionally, equipping undergraduate nursing students, utilizing teaching methods that prepare them with the necessary mental health competence for serving a wide range of populations and understanding the impact of their different illnesses and their mental health needs and health outcomes [42]. Furthermore, it necessitates ensuring that “nursing education must include engagement with, and not just fleeting exposure to, multiple perspectives on global health issues, including public health concerns and diverse cultural beliefs and practices” [43]. Despite the need to integrate mental health competencies into undergraduate nursing education, challenges such as limited faculty expertise, inadequate resources, insufficient clinical placements, the stigma surrounding mental health, transitioning from hospital to community-based care, changes in caregiver training, staff retention, and incorporating technology in mental health practice may pose barriers [15]. It is essential to further explore how mental health competencies are integrated into nursing education and the associated challenges to inform future practice. Therefore, this scoping review focuses on exploring the existing literature on integrating mental health competencies in nursing education and the associated challenges.

## Methods

This is a scoping review, which is a method used to systematically map and summarize existing literature, providing a broad overview of the extent, range, and nature of research activity in a particular field [44]. This scoping review of peer-reviewed published articles was conducted in line with the framework outlined by Arksey and O’Malley [45], and conducted by one of the researchers and a librarian. The literature was systematically searched and reported following the PRISMA extension for scoping reviews (PRISMA-ScR) reporting tool [46]. The review question was formulated considering Population, Concept, and Context [47]. This scoping review population is limited to undergraduate nursing students, while the concepts is mental health competencies and context is nursing education. There were no geographic or contextual restrictions in this scoping review. A total of 717 articles were identified (CINAHL = 30, MedLine = 48, Scopus = 282, Science Direct = 89, and Emarald Insight = 268). The literature search was conducted in November 2023. The review question is: “What is the state of evidence on strategies, pedagogical approaches, and challenges in integrating mental health competencies into undergraduate nursing education?”

### Search strategy

The librarian contributed to identifying the appropriate databases, keywords, and search strings to ensure a comprehensive search. Constant communication was maintained with the librarian to ensure that the search process went smoothly. The literature search was conducted in October 2023 after several piloting searches. The piloting searches allowed for refinement to suit the scoping review. The following databases were searched: CINAHL, MedLine “PubMed”, Scopus, Emerald Insight, and Science Direct for peer-reviewed articles related to the subject. The search strategy used keywords such as (‘integration’) AND (‘mental health competencies’ OR ‘psychiatric competencies’ OR ‘mental health skills’) AND (‘undergraduate education programs’ OR ‘undergraduate nursing education curriculum’) AND (‘challenges’ OR ‘barriers’). Table 1 shows the inclusion and exclusion criteria for article selection.

**Table 1:** Inclusion and exclusion criteria

Inclusion criteria	Exclusion criteria
Original articles	Papers that were narrative reviews, scoping reviews, systematic reviews, commentaries, dissertations, conference proceedings, editorial and book chapters
Papers published in the English language	Papers published in languages other than English
Papers focusing on integration and associated challenges of Mental Health Competencies in undergraduate Nursing Education	Papers focusing on other aspects of nursing education e.g. graduate or postgraduate programs, because they do not align with the scoping review’s objectives.
Full-text papers published between 2013–2023 and in accordance with the search keywords.	Papers published before 2013

### Screening and selection process

**Fig. 1:**
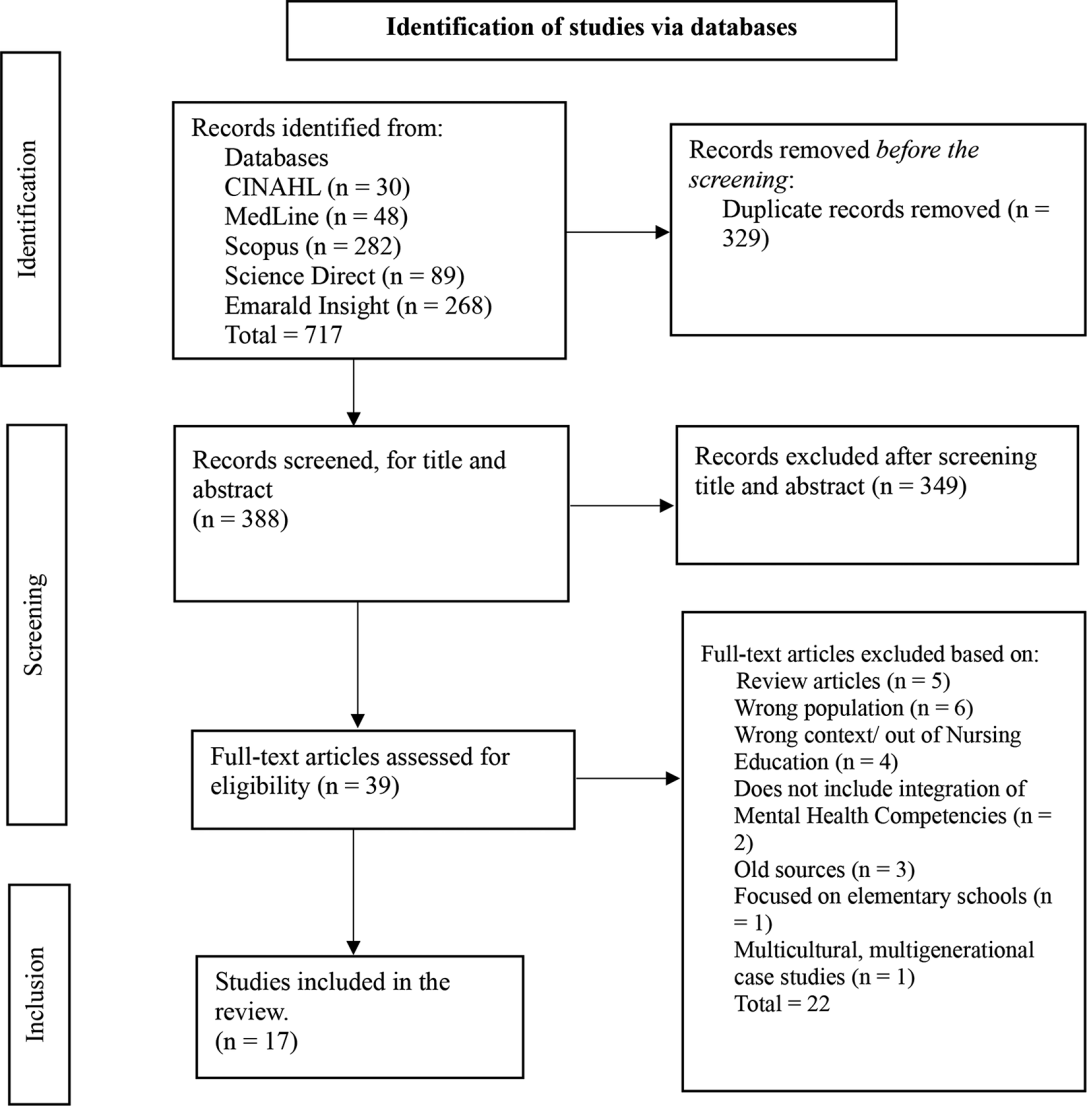
Flow chart of the review screening process

Two reviewers independently screened the titles and abstracts of the identified articles to ensure rigor and reduce potential bias. Each reviewer assessed the articles using the predefined inclusion and exclusion criteria to ensure consistency and reduce bias. Any disagreements that arose during this process, were resolved through discussion and consensus among the reviewers. In a situation where a consensus was not reached, a third reviewer was consulted to make the final decision. The screening and selection process was conducted in the following phases: firstly, the search results were imported into EPPI reviewer software. Duplicates were removed using the software’s built-in function. Secondly, carefully screening all imported studies’ titles and abstracts based on the inclusion criteria. Thirdly, the reference list of the identified articles was screened to foster a comprehensive coverage of evidence, i.e. backward snowballing [45, 48]. The titles and abstracts of the identified articles were screened for relevance by the researchers. Any discrepancies were duly resolved. Full-text screening was conducted for all the identified articles (See Fig. 1 for the review screening process flow chart). The research team scrutinized this process.

### Data extraction, analysis, and report

The following basic information regarding the eligible studies was systematically extracted and tabulated to ensure consistency: authors and publication year, title, journal, location, study design or method, data source, data analysis, and key findings (See Table 2 below for the basic information on eligible studies). The data extraction process was carried out systematically by two reviewers who collaborated to ensure consistency and accuracy. Prior to the review, the reviewers jointly developed and finalized the data extraction table, agreeing on its contents to standardize the process (See Table 2 below for basic information on the eligible studies). A third reviewer critically assessed and validated the data extraction table. In conducting scoping reviews, authors should extract only the data items that align with the scoping review questions [49]. Given the heterogeneity of the included studies, a narrative synthesis approach was used to compile, summarize, and organize the data [45, 50]. This process converted quantitative data into qualitative descriptions, identifying patterns and recurring themes [50]. A narrative synthesis typically provides a broad overview of the existing literature, identifies vital concepts, and highlights research gaps; hence best for this scoping review. The data analysis process was validated through multiple rounds of review by the research team to ensure consistency and alignment with the research objectives. Furthermore, a thematic analysis was conducted [45]. A thematic analysis used deductive coding to group findings into themes and subthemes. An iterative approach ensured thorough data exploration through repeated readings [51].

**Table 2:** Basic information on eligible studies

Author & Publication Year (the years were not provided due to the citation style)	Title	Journal	Location	Design or method	Data Source			Data Analysis	Key Findings
Alexander et al. [52]	Mental health simulation with student nurses: A qualitative review.	Clinical Simulation in Nursing	Australia	A qualitative study	Semi-structured focus group			Thematic analysis	The study identified challenges in teaching mental health nursing, such as the need for nontraditional teaching methods and difficulty bridging theory and practice.
Atashzadeh-Shoorideh et al. [15]	Effectiveness of implementation of “mental health nursing students’ clinical competency model” on academic performance of nursing students	F1000Research	Iran	Quantitative study (Semi experimental research)	Pre and post-intervention using a Likert scale			SPSS software-version 21.	The study revealed a significant improvement in the nursing students’ clinical competency level when the “mental health nursing students’ clinical competence model” was implemented.
Benjenk et al. [26]	Overcoming the Dual Stigma of Mental Illness and Aging: Preparing New Nurses to Care for the Mental Health Needs of Older Adults	The American Journal of Geriatric Psychiatry	USA	A qualitative study	Semi-structured, in-depth interviews			A thematic analysis approach	The study highlighted persistent mental health prejudices among the challenges hindering the integration of mental health competence into undergraduate nursing education.
Choi et al. [53]	Clinical Education in Psychiatric Mental Health Nursing: Overcoming current challenges	Nurse Education Today	South Korea	Mixed-method study	Structured questionnaires and open-ended questions			Quantitative data were analysed with IBM SPSS 22, while open-ended questions were analysed qualitatively.	Mental health competencies are integrated into undergraduate nursing education by introducing a curriculum for an undergraduate mental health nursing clinical practicum.
Felton and Wright [54]	Simulation in mental health nurse education: The development, implementation, and evaluation of educational innovation.	Nurse Education in Practice	United Kingdom (UK)	A mixed-methods study	A questionnaire which includes included both open (free text response) and Likert scale questions was used			Descriptive statistics was used to analyse quantitative data, while the qualitative data was analysed thematically	Students perceived the simulation scenarios as reflective of real-life situations encountered in mental health settings
Graham, et al. [55]	Educating the Educators: Determining the Uniqueness of Psychiatric Nursing Practice to Inform Psychiatric Nurse Education	Issues in Mental Health Nursing	Canada	A mixed method Study (Quantitative Survey design incorporating focus group)	Survey and focus group			Statistical Package for the Social Sciences and Thematic analysis of narrative survey responses and focus group transcripts	The study highlights the importance of comprehensive knowledge of mental health, mental illness, and addictions in psychiatric nursing practice.
Harmon and Hills [56]	Transforming psychiatric mental health nursing education with team-based learning.	Archives of Psychiatric Nursing	United States of America (USA)	A quantitative (An uncontrolled before and after design)	Practice exit examination scores using **student-reported Study Time**			SPSS version 22.0	Mental health competencies are integrated into undergraduate nursing education through a Team-Based Learning (TBL) instructional approach in an undergraduate Psychiatric-Mental Health (PMH) course.
İnan et al. [57]	The Impact of Mental Health Nursing Module, Clinical Practice and an Anti-Stigma Program on Nursing Students’ Attitudes toward Mental Illness: A Quasi-Experimental Study	Journal of Professional Nursing	Turkey	Quantitative quasi-experimental design	Socio-demographic Information Form, the Beliefs Toward Mental Illness Scale (BMI) and the Social Distance Scale			Statistical Package for the Social Sciences (SPSS), Version 16.0	Mental health competencies are integrated into undergraduate nursing education through the mental health nursing module and the integration of an anti-stigma program in the module in clinical practice.
Jacobs and Venter [31]	Standardised patient-simulated practice learning: A rich pedagogical environment for psychiatric nursing education.	African Journal of Health Professions Education	South African	A qualitative study	An open-ended questionnaire			A qualitative inductive method of data analysis	Simulation helps to build confidence in undergraduate nursing students in mental health
Jacobs [58]	The benefits of experiential learning during a service-learning engagement in child psychiatric nursing education.	African Journal of Health Professions Education	South Africa	A qualitative study	Structured reflection reports			A thematic analysis approach	The study identified challenges such as intercultural communication and collaboration, suggesting areas where nursing students may face difficulties integrating mental health competencies into their education.
Lockertsen et al. [16]	Second-year undergraduate nursing students’ experiences with clinical simulation training in mental health clinical practice: A focus group study.	Nurse Education in Practice	Norway	A qualitative study	Focus group interviews			Systematic text condensation.	Mental health competencies are integrated into undergraduate nursing education through the use of simulations.
Marriott et al. [59]	Nurse Educators’ Pedagogical Approaches Addressing Student Nurses’ Mental Health Care Competence: A Qualitative Study.	Issues in Mental Health Nursing	Norway	A qualitative study	Individual interviews		Inductive qualitative content analysis		Educators actively encourage nursing students emotionally, ethically, and cognitively to influence their attitudes, perspectives, and empathy.
Rahmani et al. [60]	Nurses’ experiences of the causes of their lack of interest in working in psychiatric wards: a qualitative study.	BMC Nursing	Iran	A qualitative study	Unstructured interviews		Conventional content analysis approach		Stigmatisation negatively affects nurses’ morale.
Rodríguez-Almagro et al. [61]	Level of Stigma among Spanish Nursing Students toward Mental Illness and Associated Factors: A Mixed-Methods Study	International journal of environmental research and public health	Spain	A mixed-methods study	Questionnaires and interviews		An SPSS v. 24.0 was used for the quantitative analysis, while Atlas.ti programme was used for the qualitative analysis.		Mental health competencies are integrated into undergraduate nursing education through theoretical training in psychiatry and mental health.
Shen et al. [62]	Exploring the experience of undergraduate nursing students following placement at psychiatric units in China: A phenomenological study.	Nurse Education in Practice	China	A qualitative study	Semi-structured face-to-face, in-depth interviews	Data were analysed using Colaizzi’s seven-step analysis method.			Psychiatric placements served to reduce stigmatising attitudes among nursing students, emphasising the importance of direct engagement with individuals with mental illness in changing perceptions.
Şengün et al. [63]	The Effect of a Peer Education program on nursing students’ beliefs Toward mental illnesses and their Career Choices	Perspectives in Psychiatric Care	Turkey	A quantitative study (Quasi-experimental pre- and post-test follow-up design)	Pretest, postintervention (post-test 1), and 6 months after (post-test 2) the intervention. (Questionnaire)	Quantitative data analysis using the Kolmogorov–Smirnov test. The BMI subsection’s mean scores were compared using the Friedman test and further analysis using a paired Wilcoxon test with Bonferroni correction.			Interventions such as peer education (PE) programs effectively change negative beliefs and increase interest in psychiatric nursing as a career choice among nursing students.
Terry [49]	In the middle: A qualitative study of talk about mental health nursing roles and work.	International Journal of Mental Health Nursing,	United Kingdom (UK)	A qualitative study	Individual interview	A thematic analysis approach			Findings underscore the complexity of mental health nursing roles and the challenges in articulating these roles within the healthcare system.

## Results

Altogether, 17 studies were identified that met the inclusion and exclusion criteria. The percentages representing the distribution of retrieved articles’ publication years, region of publication, and adopted research design/ methods are presented below. Table 3 above presents the Thematic table of the analysed data. Table 4 below presents the quantitative description of the eligible articles.

**Table 3:** Thematic table

Themes	Subthemes
Strategies and Pedagogical Approaches for Integrating Mental Health Competencies in Undergraduate Nursing Education	• Pedagogical Strategies and Educator-based approaches • Mental health nursing modules and interventions • Empathy, Clinical Competency, and Core Competencies in Psychiatric Nursing Practice
Challenges to integrating Mental Health Competencies in undergraduate Nursing Education	• Challenges related to instructional approaches/methods • Values/Attitudes towards Mental Illness

**Table 4:** Quantitative description of eligible articles

Years of article publication	Number (n)	Percentage (%)
2015	1	5.88%
2016	1	5.88%
2017	2	11.76%
2018	2	11.76%
2019	3	17.65%
2020	3	17.65%
2021	2	11.76%
2023	3	17.65%
**Region of article publication**	**Number (n)**	**Percentage (%)**
Canada	1	5.88%
United States of America (USA)	2	11.76%
South Africa	2	11.76%
United Kingdom (UK)	2	11.76%
Norway	2	11.76%
Iran	2	11.76%
South Korea	1	5.88%
Turkey	2	11.76%
Spain	1	5.88%
China	1	5.88%
Australia	1	5.88%
**Design/method**	**N**	**%**
Mixed method approach	4	23.52%
A qualitative study	9	52.94%
Quantitative study	4	23.52%
Total	17	100%

### Quantitative description of eligible articles

The quantitative description of the eligible articles demonstrates that 1 (5.88%) of the articles was published in the year 2015, 1 (5.88%) was published in 2016 and 2 (11.76%) were published in 2017. Furthermore, 2 (11.76%) were published in 2018, 3 (17.65%) of the articles were published in 2019, 3 (17.65%) published in 2020, 2 (11.76%) published in 2021, and 3 (17.65%) were published in 2023. The quantitative description of the eligible studies further revealed that **1 (**5.88%) of the articles was published in Canada, 2 (11.76%), were published in the United States of America (USA), 2 (11.76%) in South Africa, 2 (11.76%) in the United Kingdom (UK), 2 (11.76%) in Norway, 2 (11.76%) in Iran, 1 (5.88%) in South Korea, 2 (11.76%) in Turkey, 1 (5.88%) in Spain, 1 (5.88%) in China and 1 (5.88%) in Australia. Additionally, 4 (23.52%) of the studies adopted a Mixed method approach, 9 (52.94%) were qualitative studies while 4 (23.52%) were quantitative studies.

### Scope of the scoping review and General characteristics of the literature search

Seventeen studies met the inclusion and exclusion criteria, representing diverse geographic regions and research designs (Table 1). A quantitative description of these studies, including publication years and research methods, is provided under “Quantitative description of eligible articles”, above. The scoping review findings were grouped into two main themes: (1) Strategies and Pedagogical Approaches for Integrating Mental Health Competencies in Undergraduate Nursing Education and (2) Challenges to Integrating Mental Health Competencies in Undergraduate Nursing Education.

#### Theme 1: **Strategies and Pedagogical Approaches** for Integrating Mental Health Competencies in Undergraduate Nursing Education

This highlights the various educational methods and strategies employed to integrate mental health competencies into undergraduate nursing education programs. The findings emphasize how these approaches contribute to the preparation of nursing students with mental health competencies. Subthemes include; Pedagogical Strategies and Educator-based approaches, Mental health nursing modules and interventions and Empathy, clinical competency, and Core Competencies in Psychiatric Nursing Practice

### ***Pedagogical Strategies****and Educator-based approaches*

The active involvement of nursing educators in implementing diverse pedagogical approaches, such as person-centred methods and reflective practices, was highlighted across multiple institutions. Educators play a key role in emotionally, ethically, and cognitively supporting students, shaping their attitudes and empathy toward mental health. These efforts indicate that integrating mental health competencies is a critical focus in undergraduate nursing education. Educators ensure that students acquire factual knowledge about mental health and understand its relevance in nursing practice. This scoping review underscores the importance of integrating mental health competencies into undergraduate nursing education to equip students with the necessary skills and knowledge for future practice. Various strategies were adopted for this integration, with one paper emphasizing the active engagement of educators in using approaches such as person-centred care and reflective learning. These methods help students better understand and incorporate mental health knowledge into their clinical practice, with educators striving to foster empathy, critical thinking, and positive attitudes toward mental health care [59]. These educators are committed to ensuring that students develop factual knowledge about mental health and an understanding of the scope of nursing in the mental health context [59]. They are creative and responsive in addressing students’ learning needs. They are dedicated to preparing students to achieve mental health care competence, which implies that integrating mental health competencies is a significant focus in undergraduate nursing education [59]. Several reviewed articles reported the use of strategies such as simulation, PBL, TBL, clinical immersion, and critical reflective journaling. These approaches enhanced students’ confidence, knowledge, understanding, critical thinking, engagement, motivation, teamwork, and communication skills in dealing with patients with mental health conditions [31]. For example, Harmon and Hills [56], reported significant improvements in Psychiatric-Mental Health (PMH) exit examination scores over four semesters following the implementation of a TBL instructional approach in an undergraduate PMH course. This score increase suggests a positive impact on students’ acquisition of PMH content. In another reviewed study student participants reported that the simulation experience was relevant to their nursing practice and significantly impacted their confidence, clinical skills, and communication skills [54]. Providing simulation to enhance students’ understanding of mental health provides them with practical, real-world scenarios that enhance students’ readiness for clinical practice. Students perceived the simulation scenarios as reflective of real-life situations encountered in mental health settings [54]. Another study indicated simulation increased students’ feelings of preparedness for complex clinical situations and enhanced their coping abilities [16, 31]. Similarly, Jacobs’ [58] highlighted how service-learning experiences allow nursing students to apply theoretical knowledge in real-world community contexts. By engaging in community service, students can holistically address specific needs of the community, including mental health needs. Integrating theory into practice fosters a deeper understanding of mental health concepts and promotes the development of mental health competencies among nursing students.

### Mental health nursing modules and interventions

Specific mental health nursing modules and interventions, such as anti-stigma programs, were also effective in integrating mental health competencies into the nursing curriculum. These modules enhanced students’ knowledge and positively shifted their perceptions of mental illness, illustrating how competencies are integrated into undergraduate nursing education. This approach links the pedagogical strategies to the broader concept of integration, emphasizing that the strategy employed reflects a deep and wide-ranging incorporation of mental health competencies into the nursing curriculum. İnan et al. [57] shed light on how mental health nursing competence is integrated into undergraduate curricula through the mental health nursing module and the integration of an anti-stigma program in the module in clinical practice. The study reported a positive change in nursing student’s perception of dangerousness after the model. Through an analysis of how these interventions alter students’ attitudes and lessen stigma, the study advances our knowledge of how mental health competencies are incorporated into undergraduate nursing education. It further emphasizes how crucial nursing students are to have good attitudes fostered by exposure to patient viewpoints, practical experiences, and effective teaching approaches. Similarly, Rodríguez-Almagro et al. [61] evaluated the level of stigma toward people with mental health problems among nursing students. They found out that stigma decreases as students advance in their degrees, particularly after theoretical psychiatry and mental health training. Another study revealed the effectiveness of interventions like peer education (PE) programs in changing negative beliefs and increasing interest in psychiatric nursing as a career choice among nursing students [63]. This was used in integrating mental health competencies into undergraduate nursing education. The findings of the reviewed literature suggest that incorporating such programs into the nursing curriculum helps combat stigma and foster a positive attitude toward working in psychiatric nursing.

### Empathy, Clinical Competency, and Core Competencies in Psychiatric Nursing Practice

A significant improvement in the nursing students’ clinical competency level was reported when the “mental health nursing students’ clinical competence model” was implemented [15]. This shows that certain treatments can improve when mental health competencies are included in nursing education. Another study by Choi et al. [53] demonstrated the extent of integrating mental health competence into undergraduate nursing education by introducing a curriculum for an undergraduate mental health nursing clinical practicum. According to the reviewed article, evaluating nursing students’ learning outcomes from the introduced curriculum and exploring nursing students’ perceptions of the clinical practice revealed a significant improvement in students’ level of empathy [53]. Significantly, undergraduate students’ level of empathy may be increased due to teaching strategies such as adopting critical reflecting journalism, self-understanding activities, experimental learning, and other clinical settings, as reported by the researchers. Choi et al. [53] additionally observed that undergraduate nursing students gained an understanding that not all individuals with psychiatric conditions exhibit aggression and that these patients are capable of socializing with others. Furthermore, Graham et al. [55] identified essential competencies integrated into undergraduate nursing education for mental health assessment, health promotion, therapeutic relationship building, counselling, and behaviour management. These include the foundational values, beliefs, and attitudes that shape psychiatric nursing practice, including empathy, compassion, and person-centered care. The study by Shen et al. [62] indicated that psychiatric placements served to reduce stigmatizing attitudes among nursing students, emphasizing the importance of direct engagement with individuals with mental illness in changing perceptions.

#### Theme 2: Challenges to integrating Mental Health Competencies in undergraduate Nursing Education

This theme highlights the barriers and obstacles encountered in integrating mental health competencies into undergraduate nursing education. Sub-themes include challenges related to instructional approaches/methods and Values/attitudes towards mental illness.

### Challenges related to instructional approaches/methods

Among the significant challenges highlighted in this scoping review was the absence of standardized frameworks for mental health nursing education. In the absence of consistent guidelines or frameworks, ensuring that comprehensive mental health competencies are integrated into undergraduate nursing education, becomes challenging.Atashzadeh-Shoorideh et al. [15] highlighted variability in clinical mental health competence among undergraduate nursing students. This variability could be attributed to differences in educational approaches, clinical experiences, and levels of exposure to mental health settings. Furthermore, Terry [52, 64] reported negative public attitudes (stigma) associated with mental health nursing not only affect nurses’ morale but may also influence nursing students’ perceptions and interest in pursuing careers in mental health nursing. Additionally, concerns over patient behaviour, such as fear of patient assault and aggression, further escalate these challenges, reflecting a lack of confidence and preparedness among students. This fear may be rooted in a lack of exposure to mental health settings during their education or insufficient training in managing challenging behaviours. Furthermore, the study reported that unequal student contributions in group work activities, difficulties in understanding patients’ symptoms, and negative attitudes toward mental health consumers limit the effectiveness of specific pedagogical approaches. These challenges indicate potential gaps in nursing education programs regarding developing mental health competencies and cultivating positive attitudes toward mental health care [52].

### Values/Attitudes towards Mental Illness

The persistence of prejudice, stigma, and misconceptions about mental illness among nursing students, even after practicum experiences, was a recurring challenge [26, 53]. Shen et al. [62] revealed that issues such as stereotypical impressions of mental illness, communication difficulties, emotional challenges, and safety concerns are identified as hurdles that students encounter during their placements. These challenges pose obstacles to integrating mental health competencies in nursing education. Additionally, Terry [64] reported how mental health nurses perceive their roles as being in the middle, with a lack of clarity in their professional identity. This uncertainty may affect the teaching and learning of mental health competencies in nursing education, as students may struggle to understand the role and contribution of mental health nursing within the broader healthcare context. This suggests that while integration exists to some degree, these challenges must be addressed while incorporating mental health competencies into nursing education. Jacobs’ [58] reported that challenges such as intercultural communication and collaboration are identified, suggesting that nursing students may face difficulties integrating mental health competencies into their education. A study by Alexander et al. [52] also identified challenges in teaching mental health nursing, such as the need for nontraditional teaching methods and difficulty bridging theory and practice.

## Discussion

This scoping review provided insight into the extent to which mental health competence is integrated into nursing education and the associated challenges. The scoping review revealed that various approaches and interventions are used to integrate mental health competencies into nursing education. These approaches include teaching pedagogies such as reflection and a person-centered approach, simulation, problem-based learning, mental health nursing modules with integrated anti-stigma programs, implementing clinical competence models, and introducing curricula for undergraduate mental health nursing clinical practicums. In practice, these approaches enhance decision-making, foster therapeutic relationships, and support behaviour management which are crucial skills in mental health nursing [65]. The varieties of models and interventions revealed by the reviewed studies can be classified as mandatory or optional. Mandatory components such as integrated anti-stigma campaigns, psychiatric clinical practicums, and structured mental health nursing courses [53, 66]. Drawing on Watson’s Caring Science theory that emphasizes holistic and compassionate care, the integration of mental health competence into undergraduate nursing education reflects the theory’s focus on aligning the mind, spirit, and body with the caring science. Watson’s framework advocates for a person-centered and empathetic approach that enhances therapeutic communication and supports the establishment of therapeutic relationships necessary to mental health nursing [30]. In line with the current scoping review report, Bosse et al. [67] confirmed that incorporating Trauma-informed education practices (TIEP) into a mental health course for undergraduate nursing students is both viable and advantageous. It enables faculty members to exemplify essential skills and values intrinsic to nursing practice. Similarly, another study supported our findings and reported that simulation improves the students’ abilities in therapeutic communication, critical thinking, problem-solving, decision-making, and risk assessment within mental health nursing practice [68]. Additionally, it has been observed to reduce students’ apprehension and unease when interacting with individuals with mental health disorders, fostering increased self-assurance and comprehension of mental illness [68]. Beyond the findings of this current scoping review, the literature also highlights that Psychiatric-Mental Health Clinical Course (P-MHCC) assignments and clinical educational experiences have been used to integrate mental health competencies into undergraduate nursing education, promoting evidence-based practice [69]. Similarly, Horntvedt et al. [70] argued that incorporating diverse interactive teaching methods into course assignments has been noted to enhance undergraduate nursing students’ knowledge and evidence-based practice skills. These findings emphasize the importance of educators’ roles in mental health nursing education and the need for faculty to receive training to enhance their competency and awareness of mental health illnesses [71].

Integrating mental health competencies into undergraduate nursing education is crucial in preparing the next generation of nurses. A report demonstrated that undergraduate nursing students’ mental health competence was enhanced upon their practical performance of different modalities such as student role plays, problem-based learning of various mental health disorders, interviewing standardized patients, and concept-integrated maps from tailored scenarios [5]. Similarly, various teaching pedagogies, such as critical reflective journalism, clinical immersion, TBL, and PBL, were found to be adopted in integrating mental health competencies into undergraduate nursing education. Similar to this scoping review report, Jacobs and Venter [31] and Farooq et al. [72] reported student’s positive experiences after using simulation to integrate mental health competencies into undergraduate nursing students. Other educational interventions, such as mental health nursing modules on nursing students’ perceptions of mental illness and anti-stigma campaigns, were reported in this scoping review as integrated into undergraduate nursing education. These are regarded as mandatory components of undergraduate mental health nursing education that form the foundation of student’s training [52]. Additionally, peer education, the TBL instructional approach, virtual experiences, and virtual simulations were reportedly integrated into undergraduate nursing education. These components are regarded as optional interventions. They advance therapeutic communication skills, foster evidence-based practice, and enhance and promote empathy in healthcare [73]. Both mandatory and optional interventions are directly applicable in addressing practical needs such as enhancing practical relationships and mitigating stigmatization in mental healthcare [74]. These mental health competencies were found to be beneficial when integrated into undergraduate nursing education. Interestingly, the above findings align with existing literature [75, 76]. Further research is recommended to explore these and alternative approaches, particularly in the burgeoning area of simulation.

The scoping review findings further highlighted the improvement of nursing students’ level of empathy after introducing the learning curriculum and exploring nursing students’ perceptions of the clinical practice as a method of integrating mental health competence into undergraduate nursing education. In practice, these approaches as crucial in enhancing nurses’ ablity to foster therapeutic relationships crucial for the delivery of effective mental health. Watson’s Caring Science theory further reinforces this connection by advocating for the need for empathy as a crucial component of healing [77]. Existing literature confirmed that studies within mental health nursing have underscored the significance of empathy in fostering the relationship and therapeutic dedication between patients and nurses [78]. Moreover, the management of educational institutions should foster a culture and climate that supports open-mindedness and empathy regarding solving the problems facing mental health challenges in contemporary society and innovative thinking [71].

Further findings from the reviewed articles demonstrate the integration of a broad range of competencies into undergraduate mental health nursing education, such as building therapeutic relationships, behaviour management, and mental health assessment. Findings also indicated that nurse educators are well involved in implementing different pedagogical approaches to foster the understanding of undergraduate nursing students. In line with the findings of this scoping review, existing literature reported that incorporating guided clinical experience into the initial year of an undergraduate nursing program can foster favourable shifts in stigmatizing attitudes among novice nurses [79].

Despite the various efforts to integrate mental health competencies into undergraduate nursing education, various challenges hinder these efforts. One study’s findings underscored difficulties such as the requirement for a curriculum covering all stages of life and clinical practicum, deficiencies in faculty numbers in specialized fields like a child and geriatric psychiatry, and challenges in coordinating clinical placements. In practice, these challenges translate to gaps in undergraduate nursing students’ preparation for practice, negatively affecting their confidence and ability to provide mental health services [38]. Furthermore, this scoping review revealed that the lack of a standardized framework for integrating mental health nursing into undergraduate nursing curricula is challenging. Also, public stigmatization associated with mental health nursing negatively affects the morale of nurses, including the perception and interest of nursing students in choosing mental health nursing as a career. Existing literature suggests that undergraduate nursing students’ self-confidence and self-awareness could be enhanced through simulations with standardized patients before commencing clinical placements in mental health settings [80]. This could foster mental health competence among undergraduate nursing students. Additionally, the fear of patient behaviours, including patient aggression, poses a challenge to the integration of mental health competencies into undergraduate nursing education. Nursing students who struggle to overcome the fear associated with nursing mental health patients find it difficult to cope with their clinical placement, which negatively affects their ability to develop competency. However, existing literature [81] advanced that the mental health competence of undergraduate nursing students and other health degree students was improved through virtual online learning processes. Participants in their study showed positive improvements in attitudes and empathy toward culture, including clinical confidence toward mental health users. The authors further maintained that their findings could effectively facilitate changes among students and challenge attitudes and perceptions toward managing mental health users [81]. Research into the impact of simulated learning on these key areas of practice is recommended.

This scoping review also revealed various challenges to the integration of mental health competence into undergraduate nursing education including nursing students’ unequal contribution to group work activities, students’ negative attitudes toward people with mental illness, difficulties understanding patients, and communication difficulties. Additionally, the scoping review also revealed challenges associated with language barriers, differences in the cultural perception of mental health illnesses, variations in communication style, and stereotypical impressions of mental illness hinder the integration of mental health competencies into undergraduate nursing education. Overall, the findings of this scoping review provide a comprehensive overview of the existing literature on integrating Mental Health Competencies in undergraduate Nursing Education, including the challenges hindering its integration.

### Recommendations

Overall, this scoping review demonstrated that, to an extent, Mental health competencies are integrated into nursing education through various approaches, including teaching pedagogies, simulation, problem-based learning, and mental health nursing modules. Despite efforts, challenges such as lack of standardized frameworks, public stigmatization, faculty shortages, and fear of patient behaviours hinder integration. It is recommended that a standardized framework be developed for integrating mental health nursing competencies into undergraduate nursing curricula. This framework should encompass all life and clinical practicum stages, address faculty shortages in specialized fields, and streamline clinical placements. Furthermore, nursing education institutions should endeavour to provide educators with training and resources to enhance their awareness and knowledge of mental health illnesses, focusing on competency development and the integration of innovative teaching pedagogies. Additionally, nursing education institutions should encourage the development of empathetic skills among students through clinical practicum experiences and educational interventions. They should also promote diversity and inclusion in undergraduate nursing education to address challenges such as communication difficulties, intercultural communication, and stereotypical impressions of mental illness, encourage equal participation in group work activities, and foster an inclusive learning environment. Future research should focus on developing standardized frameworks for integrating mental health nursing into undergraduate nursing education addressing public stigmatization, and on effective educational interventions and strategies to overcome challenges in integrating mental health competencies is necessary.

### Limitations

This scoping review has several limitations. Most of the identified studies were from developed countries, which may limit the application of findings to nursing education contexts in developing or underdeveloped regions. Furthermore, the inclusion of grey literature or unpublished studies may have provided additional perspectives and insights. The inclusion of only English-language published research may have overlooked relevant research published in other languages. Additionally, the lack of inclusion of review articles may have hindered a comprehensive understanding of the research landscape, existing knowledge, and research gaps. Also, the restricting of the scoping review article to articles published between 2013–2023 may have excluded more recent studies published outside of the selected timeframe. This limitation should be considered when interpreting the findings, as this scoping review may not have captured all trends on the topic.

### Conclusion

This scoping review provides valuable insights into the extent to which mental health competencies are integrated into undergraduate nursing education and the associated challenges. By synthesizing findings from various studies, new insights are provided into key themes, trends, and challenges in this area. The findings revealed that diverse teaching approaches, such as trauma-informed education practices, problem-based learning, and reflection simulation are adopted to integrate mental health nursing into undergraduate nursing education. These educational approaches are directly linked to practical skills necessary for effective mental health nursing practice. Mandatory interventions such as structured clinical practicums and anti-stigma campaigns and optional components that amongst others, include virtual simulations and peer education are necessary for practice. However, these efforts are constantly challenged by societal stigma, patient behaviours, unequal student contributions in group work activities, and difficulties in understanding patients’ symptoms. Effective strategies for integrating mental health competencies into undergraduate nursing education identified in this scoping review are simulation techniques, problem-based learning, critical reflective journalism, clinical immersion, and the use of virtual experiences. Overall, this scoping review contributes to the growing body of knowledge on mental health education in nursing and provides a foundation, and directions, for further research and practice in this important area.

## Data Availability

The datasets used and/or analyzed during the current study are available from the corresponding author upon reasonable request.

## Declarations

### Disclaimer

The views and opinions expressed in this manuscript are those of the authors and do not intend to communicate the stance of the institution or affiliation of the authors.
